# Post-abortion family planning use, method preference, and its determinant factors in Eastern Africa: a systematic review and meta-analysis

**DOI:** 10.1186/s13643-021-01731-4

**Published:** 2021-06-09

**Authors:** Asmamaw Demis Bizuneh, Getnet Gedefaw Azeze

**Affiliations:** 1 School of Nursing, College of Health Sciences, Woldia University, P.O.Box: 400, Woldia, Ethiopia; 2 School of Midwifery, College of Health Sciences, Woldia University, P.O.Box: 400, Woldia, Ethiopia

**Keywords:** Post-abortion, Family planning, Systematic review, Meta-analysis, Eastern Africa

## Abstract

**Background:**

Utilization of post-abortion family planning is very critical to reduce high levels of unintended pregnancy, which is the root cause of induced abortion. In Eastern Africa, it is estimated that as many as 95% of unintended pregnancies occurred among women who do not practice contraception at all. Therefore, this meta-analysis aimed to assess post-abortion family planning utilization and its determinant factors in Eastern Africa.

**Methods:**

Published papers from Scopus, HINARI, PubMed, Google Scholar, and Web of Science electronic databases and grey literature repository were searched from database inception to January 30, 2020, with no restriction by design and date of publishing. We screened records, extracted data, and assessed risk of bias in duplicate. Cochrane I^2^ statistics were used to check the heterogeneity of the studies. Publication bias was assessed by Egger and Biggs test with a funnel plot. A random-effects model was calculated to estimate the pooled prevalence of post-abortion family planning utilization.

**Results:**

A total of twenty-nine cross-sectional studies with 70,037 study participants were included. The overall pooled prevalence of post-abortion family planning utilization was 67.86% (95% CI 63.59–72.12). The most widely utilized post-abortion family methods were injectable 33.23% (95% CI 22.12–44.34), followed by implants 24.71% (95% CI 13.53–35.89) and oral contraceptive pills 23.42% (95% CI 19.95–26.89). Married marital status (AOR=3.20; 95% CI 2.02–5.05), multiparity (AOR=3.84; 95% CI 1.43–10.33), having a history of abortion (AOR=2.33; 95% CI 1.44–3.75), getting counselling on post-abortion family planning (AOR=4.63; 95% CI 3.27–6.56), and ever use of contraceptives (AOR=4.63; 95% CI 2.27–5.21) were factors associated with post-abortion family planning utilization in Eastern Africa.

**Conclusions:**

This study revealed that the marital status of the women, multiparity, having a history of abortion, getting counselling on post-abortion family planning, and ever used contraceptives were found to be significantly associated with post-abortion family planning utilization.

**Supplementary Information:**

The online version contains supplementary material available at 10.1186/s13643-021-01731-4.

## Background

Post-abortion family planning is the initiation and use of family planning methods immediately after and within 48 h of an induced or spontaneous abortion or treatment of complications before fertility returns [1, 2]. The provision of family planning is important for women in the post-abortion period because fertility can return surprisingly quickly after having an abortion. Even if a woman wants to have a child immediately after an abortion, the World Health Organization (WHO) and Federation of International Gynaecology and Obstetrics (FIGO) guidelines recommend she should wait at least 6 months before getting pregnant again [2, 3]. The global estimates for the year 2017 indicate that there were 295,000 maternal deaths worldwide, with Sub-Saharan Africa and Southern Asia accounting for approximately 86% (254,000), with Eastern Africa alone accounting for roughly 542/100,000 maternal deaths [4].

Every year, more than 44 million women have been complicated with induced abortions, and of these, around 20 million women accounted for unsafe abortions. Unsafe abortion contributes to 13% of maternal deaths globally and 37 deaths per 100,000 live births in Sub-Saharan Africa (SSA). The World Health Organization (WHO) estimates that in Eastern Africa unsafe abortion accounts for one in seven maternal deaths [1, 5]. In Africa, 99% of all abortions carried out were unsafe, and the risk of maternal death from an unsafe abortion is one in every 150 procedures which is the highest in the world [6, 7].

Offering a wide range of post-abortion family planning methods is likely to increase family planning uptake; as a result, in the immediate post-abortion period, WHO recommended that a woman can safely use a full range of contraceptive methods, including condoms, spermicides, oral contraceptives, emergency contraceptive pills, injectable, implants, IUDs, and female sterilization [8]. Almost every abortion-related death and disability could be prevented through sexuality education; use of effective contraception; provision of safe, legal-induced abortion; and timely care for complications. Post-abortion family planning (PAFP) has been proposed as a key strategy to reduce unintended pregnancy, repeat-induced abortions and lower morbidity and mortality among women, neonates, infants, and children [9–11]. However, the accessibility and quality of PAFP services remain a challenge in Eastern Africa where a higher number of unintended pregnancies occur each year. In Eastern Africa, a lot of fragmented studies have been conducted to assess post-abortion family planning utilization and its associated factors among post-aborted women. These fragmented studies reported that the magnitude of post-abortion family planning utilization in Eastern Africa ranged from 15.5 to 90.6% [12–20]. From the reports of these studies, there was a great variation and inconsistency related to the prevalence of post-abortion family planning utilization throughout East African countries.

The reasons for the above variation in the prevalence and associated factors of post-abortion family planning utilization among East African women have not yet been investigated. The provision of safe, legal abortion is essential to fulfilling the global commitment to the Sustainable Development Goal (SDGs) of universal access to sexual and reproductive health (target 3.7). A systematic review and meta-analysis would help policymakers and health managers and planners to make evidence-based decisions that have taken into account all available information, as well as indicating the quality of the results. Therefore, the main aim of this systematic review and meta-analysis was to estimate the pooled prevalence of post-abortion family planning utilization and to identify its associated factors among post-aborted women that could be used in policy formulation and evidence-based decision-making practices in Eastern Africa.

## Materials and methods

### Study reporting

In this systematic review and meta-analysis, we used the “Preferred Reporting Items for Systematic Reviews and Meta-Analyses (PRISMA)” guideline [21] (Table S1).

### Databases and search strategies

In this systematic review and meta-analysis, we checked databases without the restriction of design and date of publishing. The search included keywords and MeSH terms, combinations, and snowball searching about relevant papers providing data on the prevalence of post-abortion family planning utilization and/or its associated factors in a search focused on eastern Africa. Studies were searched from databases including PubMed/MEDLINE, Web of Science, Embase, Scopus, HINARI, Science Direct, African Journals, and Cochrane Library. Besides, bibliographies of identified articles and grey literature, like Google and Google scholar, Mednar, World Wide Science, and online University repositories have been scientifically searched (Table 1). The following websites were hand-searched: Ipas, Jhpiego, Family Health International, Marie Stopes International, Population Council, Post-abortion Care Consortium, Gynuity Health Projects Engender Health, PRIME II, and Eldis. The following search terms were used: (Prevalence OR Epidemiology OR Magnitude) AND (determinants OR associated factors OR predictors) AND (Post-abortion OR postabortion OR postabortal OR post abortal OR post-abortal OR incomplete abortion OR incomplete abortions OR unsafe abortion OR unsafe abortions AND (family planning use OR family planning utilization OR family planning uptake OR family planning services OR contraceptive use OR contraceptive utilization OR contraceptive uptake OR birth control OR fertility control OR population control) AND (east Africa) and related terms. All countries are categorized under Eastern Africa, namely, Kenya, Uganda, Tanzania, Rwanda, Burundi, Ethiopia, South Sudan, Djibouti, Eritrea, Mozambique, Madagascar, Malawi, Zambia, Comoros, Mauritius, Seychelles, and Somalia. The search terms were used independently and in amalgamation using Boolean operators like “OR” or “AND” and related terms. All article searched from databases was exported to EndNote library. Systematic review with narrative synthesis was used to summarize the findings of articles in Eastern Africa.

**Table 1:** Search strategy for the MEDLINE/PubMed and Google Scholar databases to assess post-abortion family planning utilization and its associated factors in eastern Africa

Databases	Searching terms	Number of studies
MEDLINE/PubMed	(Prevalence OR Epidemiology OR Magnitude) AND (determinants OR associated factors OR predictors) AND (Post-abortion OR postabortion OR postabortal OR post abortal OR post-abortal OR incomplete abortion OR incomplete abortions OR unsafe abortion OR unsafe abortions) AND (family planning use OR family planning utilization OR family planning uptake OR family planning services OR contraceptive use OR contraceptive utilization OR contraceptive uptake OR birth control OR fertility control OR population control) AND (east Africa)	2233
Google Scholar	“Prevalence AND determinants OR associated factors AND Post-abortion AND family planning use OR family planning utilization OR family planning uptake OR family planning services OR contraceptive use OR contraceptive utilization OR contraceptive uptake AND east Africa.”	1530
From other databases		598
Total retrieved articles		4361
Number of included studies		29

### Inclusion and exclusion criteria

In this systematic review and meta-analysis, both published and unpublished articles in the English language without time limiting that reported prevalence of post-abortion family planning utilization and/or its associated factors among women in Eastern Africa were included. Articles searched from January 1–30, 2020, were included. Additionally, we restricted our search to observational studies such as cross-sectional, comparative cross-sectional, case-control, and retrospective and prospective cohort studies. Interventional studies, case reports, letters, editorials, systematic reviews, narrative reviews, policy statements, news, and inaccessible full text after two contact attempts of the corresponding author by email were excluded from the final analysis.

### Data extraction

After removing duplicates from the Endnote version X8 software, all studies were exported to a Microsoft Excel spreadsheet. Two authors (ADB and GGA) independently extracted all important data using a standardized data extraction form which was adapted from the JBI data extraction format. Substantial agreement between reviewers, i.e., Cohen’s kappa coefficient >0.60, was accepted and resolved through discussion and consensus. For the first outcome (prevalence), the data extraction format included (primary author, year of publication, country, study area, sample size, and prevalence with 95% CI). Data were extracted with a 2 by 2 table format, and then, the log odds ratio for each factor was calculated for the second outcome (associated factors).

### Measurement of outcomes

This meta-analysis had two outcomes. The first outcome of this study mainly focused on the prevalence of post-abortion family planning utilization in Eastern Africa. The prevalence was calculated by dividing the number of women who used post-abortion family planning by the total number of women who have been included in the study (sample size) multiplied by 100. The second outcome of the study was factors associated with post-abortion family planning utilization, which were measured by using the adjusted odds ratio from primary published studies.

### Quality evaluation

Two authors (ADB and GGA) independently assessed the quality of each study using Newcastle-Ottawa scale (NOS) for cross-sectional studies [22]. All included articles were cross-sectional in design. The methodological quality of the study, comparability of the study, and the outcome and statistical analysis of the study were the three major assessment tools we used to declare the quality of the study. Lastly, studies that scored a scale of ≥ 7 out of 10 were considered as achieving high quality. During quality appraisal of the articles, any discrepancies between the two authors were resolved.

### Data synthesis and statistical analysis

We pooled the overall prevalence estimates of post-abortion family planning utilization using the random-effects model [23]. After extraction of the articles in Microsoft Excel spreadsheet format, the analysis was carried out using the STATA version 14 statistical software. Cochrane Q test and *I*^2^ statistics were computed to assess heterogeneity among studies [23, 24]. After computing the statistics, the results showed there was significant heterogeneity among the studies (*I*^2^ = 99.2%, p <0.001); therefore, considerable heterogeneity was assumed, and Mantel-Haenszel random-effects model meta-analysis was employed to estimate the Dersimonian and Laird’s pooled effect [25]. Publication bias was also assessed using Egger’s correlation and Begg’s regression intercept tests at a 5% significance level [26, 27]. Due to the presence of publication bias in the study, Egger’s test was statistically significant (p=0.006); as a result, trim-fill analysis was executed. Subgroup analysis was done by the study country, sample size, and year of publication to minimize the random variations between the point estimates of the primary study. Forest plot format was used to present the pooled point prevalence with 95% CI. For associations, a log odds ratio was used to decide the association between associated factors and post-abortion family planning utilization.

## Results

**Fig. 1:**
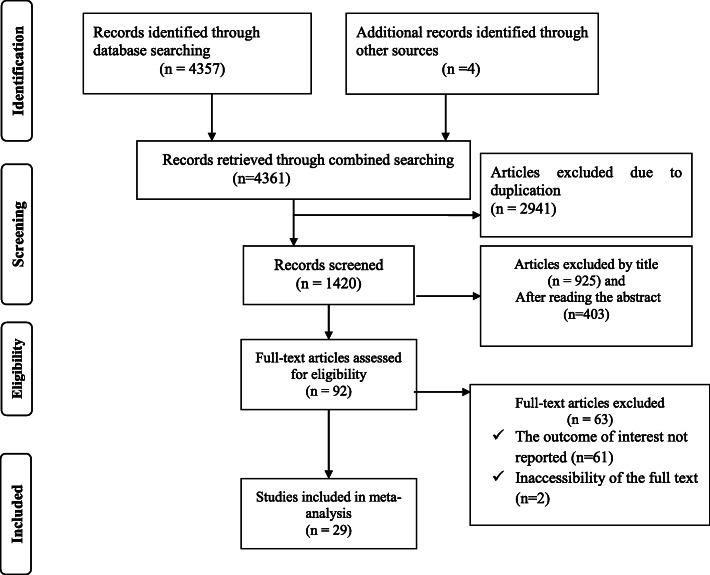
Flow chart of selection for systematic review and meta-analysis of post-abortion family planning utilization and its associated factors in eastern Africa

Of the total retrieved articles from different databases, 1420 articles remained after expunging the duplicates. Out of the remaining articles, 925 and 403 were excluded after reviewing the titles and abstracts, respectively. Therefore, 92 full-text articles were accessed and assessed for eligibility based on the preset criteria, which resulted in the further exclusion of 63 articles primarily due to the outcome of interest not reported (*n*=61) and inaccessibility of the full text (*n*=2). As a result, 29 studies meeting the eligibility criteria were included in the final meta-analysis (Fig. 1).

### Characteristics of original studies

Among the 29 articles which were published in East African countries until January 2020, 70,037 study participants were involved to determine the pooled prevalence of post-abortion family planning utilization. Regarding the study design, all studies were cross-sectional. Fifteen of the studies were from Ethiopia [15–18, 28–38], five studies were from Kenya [20, 39–42], four studies were from Tanzania [43–46], and the rest five studies were from Malawi [47], Mozambique [48], Zimbabwe [49], Somalia [50], and Rwanda [51]. Regarding the quality scores, the quality score of each original study ranged from a low of six to a high of eight (Table 2).

**Table 2:** Study characteristics included in the systematic review and meta-analysis on post-abortion family planning utilization and its associated factors in Eastern Africa

Authors	Country	Publication year	Study design	Sample size	Prevalence	Quality
Abamecha et al. [15]	Ethiopia	2016	Cross-sectional	399	72.93	7
Abebe et al. [18]	Ethiopia	2019	Cross-sectional	125	84.00	8
Alemayehu et al. [38]	Ethiopia	2009	Cross-sectional	2231	78.26	7
Asrat et al. [16]	Ethiopia	2018	Cross-sectional	552	90.58	7
Chukwumalu et al. [50]	Somalia	2017	Cross-sectional	1111	85.96	8
Erko et al. [37]	Ethiopia	2016	Cross-sectional	184	70.11	8
Evens et al. [20]	Kenya	2014	Cross-sectional	283	15.55	8
Gallo et al. [48]	Mozambique	2004	Cross-sectional	332	37.05	7
Hagos et al. [36]	Ethiopia	2018	Cross-sectional	409	70.90	8
Kassahun [35]	Ethiopia	2017	Cross-sectional	459	68.85	7
Kokeb et al. [34]	Ethiopia	2015	Cross-sectional	414	59.18	8
Lema and Mpanga [47]	Malawi	2000	Cross-sectional	464	80.39	7
Mahomed et al. [49]	Zimbabwe	1997	Cross-sectional	1009	91.97	7
Makenzius et al. [42]	Kenya	2018	Cross-sectional	810	75.19	8
Mekuria et al. [33]	Ethiopia	2019	Cross-sectional	400	78.50	8
Moges et al. [17]	Ethiopia	2018	Cross-sectional	400	61.50	7
Muche et al. [32]	Ethiopia	2019	Cross-sectional	371	45.82	7
Mutua et al. [41]	Kenya	2019	Cross-sectional	2568	55.45	9
Onyango et al. [40]	Kenya	2010	Cross-sectional	403	30.52	8
Packer et al. [51]	Rwanda	2019	Cross-sectional	68	70.59	8
Prata et al. [31]	Ethiopia	2011	Cross-sectional	1200	77.67	7
Rasch et al. [46]	Tanzania	2004	Cross-sectional	788	89.85	8
Rasch et al. [45]	Tanzania	2008	Cross-sectional	392	88.52	7
Samuel et al. [30]	Ethiopia	2016	Cross-sectional	44682	80.68	8
Seid et al. [29]	Ethiopia	2012	Cross-sectional	282	47.52	8
Solo et al. [39]	Kenya	1999	Cross-sectional	319	48.28	7
Stephens et al. [44]	Tanzania	2019	Cross-sectional	8230	80.63	8
Tesfaye and Oljira [28]	Ethiopia	2013	Cross-sectional	400	56.50	8
Wanjiru et al. [43]	Tanzania	2007	Cross-sectional	752	69.81	8

### Post-abortion family planning utilization in eastern Africa

**Fig. 2:**
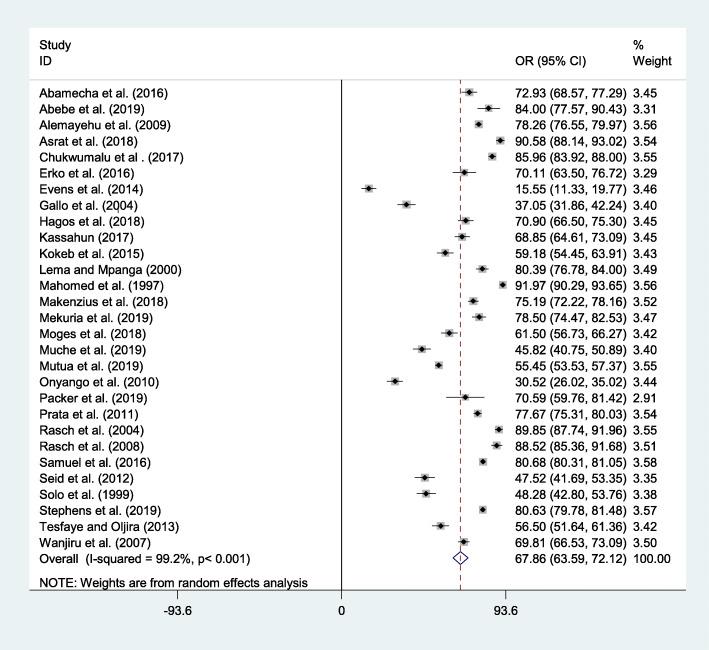
Forest plot of the pooled prevalence of post-abortion family planning utilization in Eastern Africa

In this systematic review and meta-analysis, the overall pooled prevalence of post-abortion family planning utilization was 67.86% (95% CI 63.59–72.12) (Fig. 2).

### Heterogeneity and publication bias

**Fig. 3:**
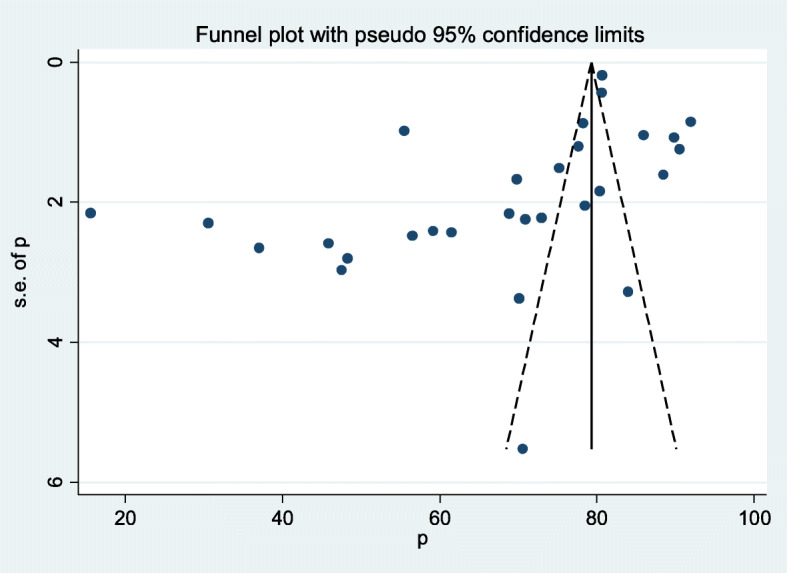
Funnel plot with 95% confidence limits of the pooled prevalence of post-abortion family planning utilization in Eastern Africa

The existence of heterogeneity within the included studies was declared with the evidence of I^2^ = 99.2%. The evidence of asymmetric distribution of the funnel plot and the statistically significant (*p*=0.006) value of Egger’s test showed the presence of publication bias (Fig. 3).

### Trim-fill analysis

**Fig. 4:**
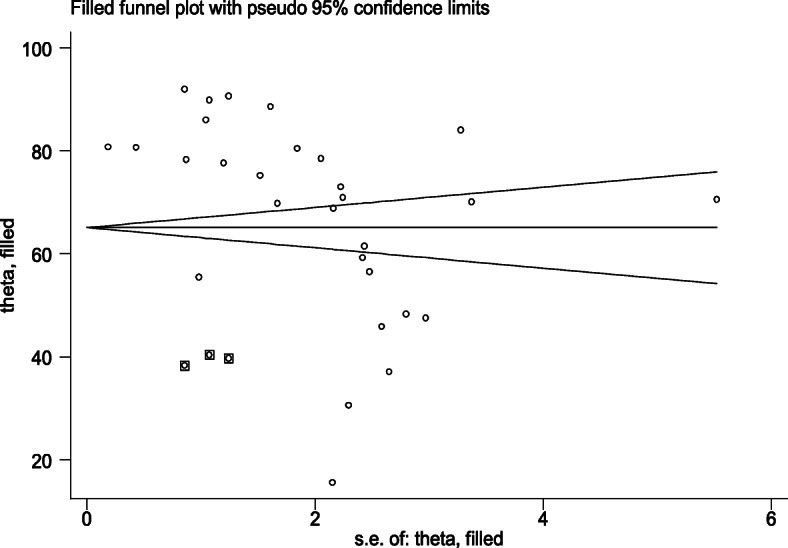
Trim-fill analysis filled funnel plot with 95% confidence limits of the pooled prevalence of post-abortion family planning utilization in Eastern Africa

In this meta-analysis, due to the presence of publication bias, we executed a trim-fill analysis by using a random-effects model; the filled meta-analysis results showed that three studies were filled, which increases the number of studies from 29 to 32 with the pooled estimate for post-abortion family planning utilization in Eastern Africa was 65.08% (95% CI 59.42–70.73, *p*<0.0001) (Fig. 4).

### Subgroup analysis

Due to the presence of heterogeneity within the included studies, a subgroup analysis based on the country, year of publication, and the sample size was conducted to identify the source of heterogeneity. Accordingly, the highest prevalence was observed in Zimbabwe with 91.97% (95% CI 90.29–93.65), and the lowest prevalence was observed in Mozambique with 37.05% (95% CI 31.86–42.24). Regarding sample size, the highest prevalence was observed in studies with a sample size of ≥1000 with 78.68% (95% CI 73.42–83.93).

Besides, we also executed a subgroup analysis based on publication year. Accordingly, the highest prevalence of post-abortion family planning utilization has occurred in studies published before 2006, which was 69.70% ((95% CI 53.83–85.58), I^2^ = 99.3, *p* < 0.001) (Table 3).

**Table 3:** Subgroup prevalence of post-abortion family planning utilization in eastern Africa

Variables	Characteristics	Included studies	Number of study participants	Prevalence with (95% CI)	I^2^, P-value
Publication year	2006 and above	24	67,125	67.42 (62.85–71.98)	99.2, <0.001
	Before 2006	5	2912	69.70 (53.83–85.58)	99.3, <0.001
Sample size	≥ 1000	7	61,031	78.68 (73.42–83.93)	99.3, <0.001
	< 1000	22	9006	64.21 (55.56–72.86)	99.0, <0.001
Country	Ethiopia	15	52,508	69.75 (65.08–74.43)	97.9, <0.001
	Kenya	5	4383	45.04 (26.05–64.04)	99.4, <0.001
	Tanzania	4	10,162	82.25 (75.35–89.15)	97.7, <0.001
	Malawi	1	464	80.39 (76.78–84.00)	-
	Rwanda	1	68	70.59 (59.76–81.42)	-
	Somalia	1	1111	85.96 (83.92–88.00)	-
	Mozambique	1	332	37.05 (31.86–42.24)	-
	Zimbabwe	1	1009	91.97 (90.29–93.65)	-
Overall		29	70,037	67.86 (63.59–72.12)	99.2, <0.001

### Types of post-abortion family planning methods utilized

In this meta-analysis, women in post-abortion time utilized the common post-abortion family planning methods, namely, injectable 33.23% (95% CI 22.12–44.34), implants 24.71% (95% CI 13.53–35.89), oral contraceptive pills 23.42% (95% CI 19.95–26.89), intrauterine devices 9.12% (95% CI 5.36–12.88), and condom 7.43% (95% CI 5.17–9.69) (Table 4).

**Table 4:** Pooled prevalence of post-abortion family planning methods among women in eastern Africa

Type of post-abortion family planning methods	Pooled prevalence 95%	I-squared
Injectable	33.23 (22.12–44.34)	99.8, p<0.001
Implants	24.71 (13.53–35.89)	99.8, p<0.001
Oral contraceptive pills (OCP)	23.42 (19.95–26.89)	97.5, p<0.001
Intrauterine devices (IUD)	9.12 (5.36–12.88)	99.0, p<0.001
Female condom	7.43 (5.17–9.69)	98.2, p<0.001
Female sterilization	0.35 (0.19–0.89)	95.2, p<0.001

### Sensitivity analysis

We executed a leave-one-out sensitivity analysis to further investigate the potential source of heterogeneity observed in the pooled prevalence of post-abortion family planning utilization in Eastern Africa. Our sensitivity analysis suggested that our findings were robust and not dependent on a single study. The pooled estimated prevalence varied between 66.96 (62.59–71.33%) and 69.78% (66.00–73.57%) for post-abortion prevalence after the deletion of a single study (Table 5).

**Table 5:** Sensitivity analysis of prevalence for each study being omitted with 95% CI prevalence of post-abortion family planning methods in eastern Africa

Study omitted	Prevalence	95% CI
Abamecha et al. [15]	67.67	63.31–72.03
Abebe et al. [18]	67.30	62.95–71.65
Alemayehu et al. [38]	67.46	62.95–71.96
Asrat et al. [16]	67.02	62.65–71.39
Chukwumalu et al. [50]	67.18	62.75–71.61
Erko et al. [37]	67.78	63.43–72.12
Evens et al. [20]	69.78	66.00–73.57
Gallo et al. [48]	68.95	64.75–73.15
Hagos et al. [36]	67.74	63.38–72.10
Kassahun [35]	67.82	63.46–72.17
Kokeb et al. [34]	68.16	63.84–72.48
Lema and Mpanga [47]	67.39	63.02–71.77
Mahomed et al. [49]	66.96	62.59–71.33
Makenzius et al. [42]	67.58	63.19–71.97
Mekuria et al. [33]	67.47	63.09–71.84
Moges et al. [17]	68.08	63.75–72.41
Muche et al. [32]	68.64	64.38–72.89
Mutua et al. [41]	68.34	64.27–72.41
Onyango et al. [40]	69.21	65.13–73.28
Packer et al. [51]	67.77	63.43–72.11
Prata et al. [31]	67.49	63.06–71.91
Rasch et al. [46]	67.04	62.65–71.43
Rasch et al. [45]	67.10	62.73–71.47
Samuel et al. [30]	67.31	61.58–73.04
Seid et al. [29]	68.56	64.28–72.84
Solo et al. [39]	68.54	64.26–72.82
Stephens et al. [44]	67.33	62.25–72.42
Tesfaye and Oljira [28]	68.26	63.95–72.57
Wanjiru et al. [43]	67.78	63.41–72.15

### Factors associated with post-abortion family planning utilization

#### Association between marital status and post-abortion family planning utilization

**Fig. 5:**
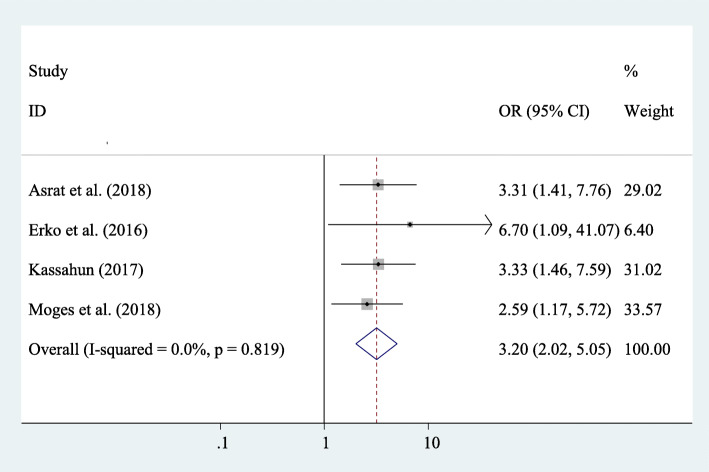
The overall pooled odds ratio of the association between marital status and post-abortion family planning utilization in Eastern Africa

In this meta-analysis, four studies were included to see the association between marital status and post-abortion family planning utilization. Those women who had married marital status were 3.2 times more likely to use family planning during the post-abortion period compared to their counterparts (AOR=3.20; 95% CI 2.02–5.05) (Fig. 5).

#### Association between parity and post-abortion family planning utilization

**Fig. 6:**
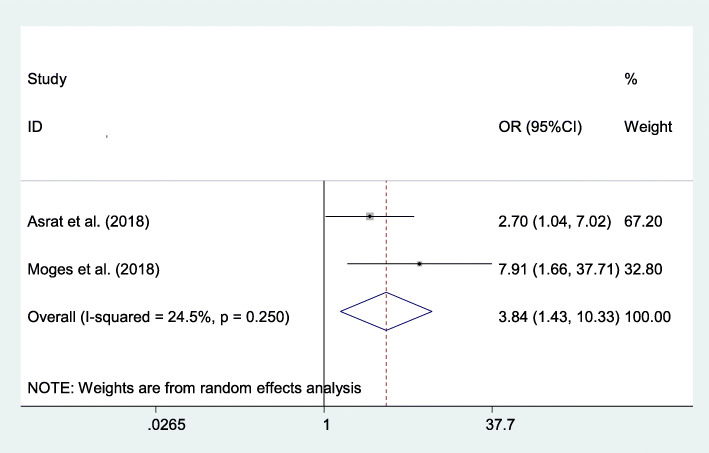
The overall pooled odds ratio of the association between parity and post-abortion family planning utilization in Eastern Africa

Two studies also indicated that multiparity was strongly associated with post-abortion family planning utilization. Multiparous women were 3.84 times more likely to use family planning during the post-abortion period compared to their counterparts (AOR=3.84; 95% CI 1.43–10.33) (Fig. 6).

#### Association between having a history of abortion and post-abortion family planning utilization

**Fig. 7:**
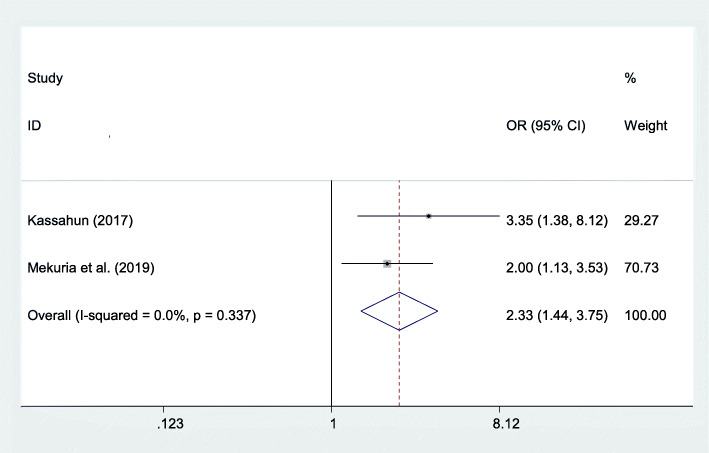
The overall pooled odds ratio of the association between history of abortion and post-abortion family planning utilization in Eastern Africa

Two studies also indicated that the history of abortion was strongly associated with post-abortion family planning utilization. Those women who had a history of abortion were 2.33 times more likely to utilize family planning during the post-abortion period compared to their counterparts (AOR=2.33; 95% CI 1.44–3.75) (Fig. 7).

#### Association between getting counselling on post-abortion family planning and its utilization

**Fig. 8:**
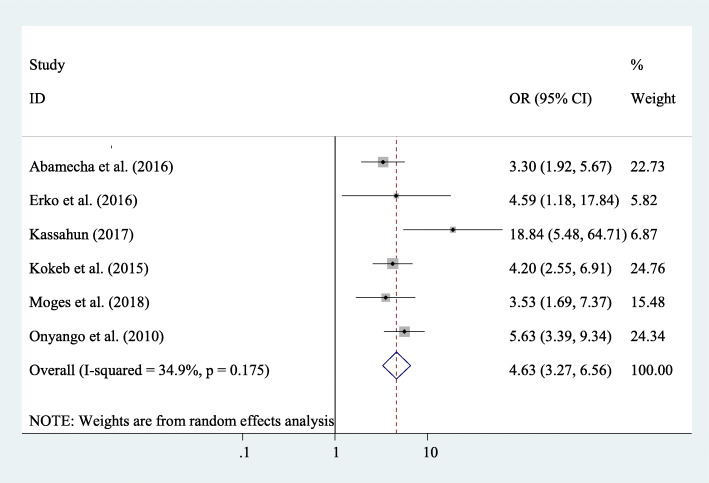
The overall pooled odds ratio of the association between post-abortion contraceptive counselling and post-abortion family planning utilization in Eastern Africa

Furthermore, eight study results from the meta-analyses of the study (Fig. 8) have also revealed that getting counselling about post-abortion family planning was a significant factor associated with post-abortion family planning utilization of women. Women who had got counselling on post-abortion family planning were 4.63 times more likely to use family planning compared to their counterparts (AOR=4.63; 95% CI 3.27–6.56) (Fig. 8).

#### Association between ever used contraceptives and post-abortion family planning utilization

**Fig. 9:**
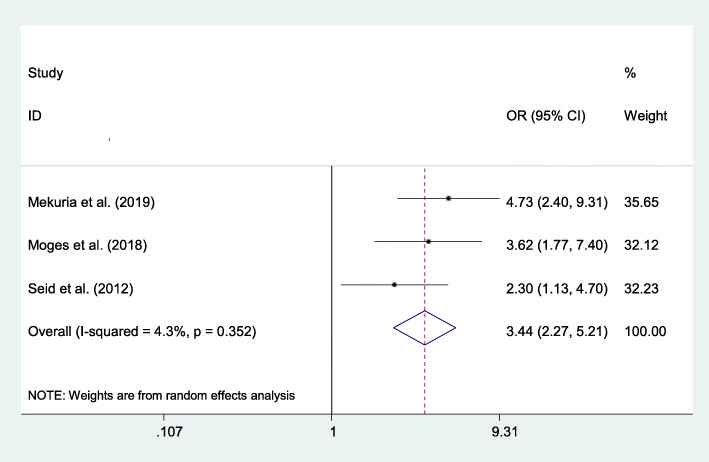
The overall pooled odds ratio of the association between ever used contraceptives and post-abortion family planning utilization in Eastern Africa

In this meta-analysis, three study results revealed that ever used contraceptive method was a significant factor associated with post-abortion family planning utilization. Women who had ever used contraceptive methods were 3.44 times more likely to use family planning compared to those women who had not used contraceptive methods (AOR=4.63; 95% CI 2.27–5.21) (Fig. 9).

## Discussions

Low post-abortion family planning utilization is considered as one of the primary and major causes of induced abortion or spontaneous abortion or stillbirth since most post-abortion women are at risk of pregnancy almost immediately. Therefore, this systematic review and meta-analysis aimed to estimate the pooled prevalence of post-abortion family planning utilization and its associated factors in Eastern Africa. In this meta-analysis, the overall pooled prevalence of post-abortion family planning utilization in Eastern Africa was 67.86% (95% CI 63.59–72.12). This is lower than the study done in Brazil 97.4% [52], Asia and Sub-Saharan Africa (SSA) 77% [53], Pakistan 73% [54], and India 81% [55]. However, it is higher than a study done in Kenya 60.9% [56] and Nepal 49.5% [57]. This might be due to variation in sample size and differences in socioeconomic status, sociocultural values, norms, religious beliefs, and study setting of the study populations. Besides, it might be due to differences in post-abortion counselling practices, availability of family planning methods and services, and more than half of the included studies in the final meta-analysis were from Ethiopia.

Regarding the subgroup analysis, the highest prevalence was observed in Zimbabwe with 91.97% (95% CI 90.29–93.65), and the lowest prevalence was observed in Mozambique with 37.05% (95% CI 31.86–42.24). The possible justification could be due to the difference in the participant’s level of awareness, educational level, religious beliefs, and various misconceptions and large gaps in the availability and distribution of facilities with basic and comprehensive post-abortion care capabilities across countries. Besides, in Zimbabwe, there is a strong family planning program with one of the highest contraceptive prevalence rates in Sub-Saharan Africa (SSA). Zimbabwe also has a restrictive abortion law, with legal abortion limited to circumstances of rape, incest, fetal impairment, or to save the woman’s life [17, 58].

In this study, the overall post-abortion family planning utilization was highest in studies published before 2006, which was 69.70% compared to studies published in 2006. The probable reason might be only five studies with a small sample size that were published before 2006 were included in the analysis, which might contribute to the higher utilization of post-abortion family planning methods. Additionally, those published articles before 2006 also include studies done in Zimbabwe which has one of the lowest abortion rates in Sub-Saharan Africa, likely due to high contraceptive use and a robust family planning program [59].

Regarding the type of post-abortion family planning methods utilized, the most commonly utilized post-abortion family planning was injectable 33.23% (95% CI 22.12–44.34), implants 24.71% (95% CI 13.53–35.89), oral contraceptive pills 23.42% (95% CI 19.95–26.89), intrauterine devices 9.12% (95% CI 5.36–12.88), and condom 7.43% (95% CI 5.17–9.69). This is in line with a study conducted in Brazil, Pakistan, India, and Nepal [52, 54, 55, 57]. This might be due to most women preferring to use short-acting methods to conceive after a short period due to higher pregnancy desire. Moreover, it might be due to provider bias towards specific methods, general demand for short-term methods (including the barriers women face in accessing longer-term methods like health care coverage, ongoing source of care, quality of care, disparate access to health information, contraception myths, and increased apprehension of side effects), and supply-related concerns might also contribute.

Women who were married were more likely to utilize post-abortion family planning compared to single women. This finding is consistent with the study conducted in Gondar, Ethiopia [60]. This might be due to married women may be likely to be having sex more regularly than unmarried women, which may explain their high post-abortion family planning utilization. Besides, currently, married women’s decision-making power on family planning has been raised [61, 62] and contraceptive prevalence continues to increase [63]. Similarly, multiparous women were more likely to utilize post-abortion family planning compared to their counterparts. This might be due to multiparous women who were at higher risk of death due to recurrent abortion, anemia, diabetes mellitus, and other chronic diseases; as a result, they decided to use post-abortion family planning for the recommended period before getting pregnant again. Moreover, multiparous women might want to limit their number of children. Additionally, the multiparous mother may feel more confident to decide on post-abortion family planning individually and by discussing with her partner.

The odds of post-abortion family planning utilization were higher among those women who had a history of abortion compared to those women who had not a history of abortion. This might be due to women who had a history of abortion may get counselling on family planning methods, and they became awarded on the use of post-abortion family planning methods. Similarly, the odds of post-abortion family planning utilization were higher among those women who got counselling on family planning methods as compared with their counterparts. This might be explained that women who get counselling about family planning methods may easily understand the risks of frequent pregnancy for women and the growing fetus, which ultimately increases post-abortion family planning utilization.

Post-abortion family planning utilization was higher among women who used contraceptives compared to those women who never used any contraceptives. This finding is supported by a study conducted in Pakistan [54]. This might be due to women who ever used contraceptives had previous exposure to family planning services, which might influence the awareness of women towards post-abortion family planning utilization. Besides, there is limited evidence contributing to each pooled odds ratio (OR) result in the final meta-analysis.

### Limitations of the study

The study designs for all primary articles incorporated in this review were cross-sectional; as a result, the confounding variables most of the time might affect the outcome variable. Furthermore, only papers published in English were included in the review. Most of the publications were from a few countries in eastern Africa which may not be representative of the subregion, and there is also a limited sample size from some countries which makes it difficult to conclude for the entire population of the country. Lastly, relevant research published in another language, or not indexed in the selected databases, has been excluded.

## Conclusion

This study revealed that the marital status of the women, multiparity, having a history of abortion, getting counselling on post-abortion family planning, and ever used contraceptives were found to be significantly associated with post-abortion family planning utilization. Therefore, based on the study findings, the authors recommended that policies and protocols should be updated to eliminate barriers such as the requirement that women and adolescents have to be married or have parental or spousal consent for contraceptive services. Advocacy is needed from policymakers and governments for ensuring quality post-abortion family planning services and reducing the unmet need for family planning by giving individualized and patient-centred post-abortion family planning counselling and client interaction, upgrading clinical skills on post-abortion contraceptive methods and implementing efforts to reduce stigma. Generally, health systems and providers in Eastern Africa need support to ensure quality PAC in the face of a reportedly high burden of complications arising from unsafe abortion in the subregion. There is a disturbing lack of evidence on PAFP utilization in most countries in the subregion. As a result, little is known about the utilization of PAFP services in the majority of Eastern African countries. Research with longer follow-up with women, a more rigorous study design with more qualitative support to understand women’s reasons for or objections to PAFP, is needed to fill these knowledge gaps.

## Supplementary Information

**Additional file 1:.** Table S1 PRISMA 2009 Checklist

## Data Availability

The dataset supporting the conclusions of this article is available from the authors on request.

## Abbreviations

ANC: Antenatal care
CI: Confidence interval
CSA: Central Statistical Agency
EDHS: Ethiopian Demographic and Health Survey
EMDHS: Ethiopian Mini Demographic and Health Survey
OR: Odds ratio
PAFP: Post-abortion family planning
WHO: World Health Organization
